# Long fusion correction of degenerative adult spinal deformity and the selection of the upper or lower thoracic region as the site of proximal instrumentation: a systematic review and meta-analysis

**DOI:** 10.1136/bmjopen-2016-012103

**Published:** 2016-11-15

**Authors:** Xin Fu, Xiao-Lei Sun, Jonathan A Harris, Sun-Ren Sheng, Hua-Zi Xu, Yong-Long Chi, Ai-Min Wu

**Affiliations:** 1 Department of Orthopaedics, Tianjin Hospital, Tianjin, China; 2 Division of Globus Medical, Musculoskeletal Education and Research Centre (MERC), Audubon, Pennsylvania, USA; 3 Department of Orthopaedics, Second Affiliated Hospital of Wenzhou Medical University, Zhejiang Spinal Research Centre, Wenzhou, Zhejiang, China

**Keywords:** Adult spinal deformity, Adult scoliosis, Long fusion correction, Systematic review, Meta-analysis

## Abstract

**Objective:**

The aim of this study was to compare outcomes when the upper and lower thoracic regions were used as the site of proximal instrumentation to treat adult spinal deformity.

**Methods:**

MEDLINE, Embase and Cochrane library searches were performed to identify studies that compared outcome measures when the upper and lower thoracic vertebrae (UTV and LTV, respectively) were used as the site of proximal instrumentation. The weighted mean difference (WMD) was calculated for continuous outcomes, and the relative risk (RR) was calculated for dichotomous outcomes.

**Results:**

Seven articles (n=554 patients) met the final inclusion criteria, and we compared the outcome measures of a long fusion extending to the upper and lower thoracic regions. The pooled analysis revealed that extending fixation into the upper thoracic region decreased the risk of proximal junctional kyphosis (PJK) revision surgery (RR: 0.36, 95% CI 0.14 to 0.90, p<0.05). The operation time (WMD: 0.93, 95% CI 0.48 to 1.39, p<0.05) and estimated blood loss (WMD: 0.59, 95% CI 0.33 to 0.85, p<0.05) were significantly greater in the UTV group than in the LTV group. No significant differences were found in the Scoliosis Research Society pain, self-image, function, mental health, subtotal, satisfaction or total scores; the total number of complications or the total number of revision surgeries.

**Conclusions:**

Long posterior fixation extending into the upper thoracic region reduces the incidence of revision surgery related to PJK; however, it increased the operative level resulting in a longer operative time and greater estimated blood loss. This initial analysis indicates that extending fixation to the upper thoracic region is appropriate for patients who are likely to develop PJK following initial scoliosis correction.

**Strengths and limitations of this study**

- This is the first meta-analysis to compare the efficacy and safety of the upper and lower thoracic vertebrae (UTV and LTV, respectively) as the upper instrumented vertebra for correction of degenerative adult spinal deformity.
- The quality of each included study was assessed by methodological index for non-randomised studies and with high scores.
- No obvious publication bias was observed by Begg's and Egger's tests.
- Most of the pooled results showed good consistency (low heterogeneity among studies).
- No randomised controlled studies were found in this review, and there was no consistent definition of which vertebra constituted UTV or LTV among studies.

## Introduction

The global incidence of adult spinal deformity (ASD) is increasing as the elderly population grows. When non-operative treatment fails, ASD patients require surgical intervention.^1^ The main goals of surgical treatment for ASD are decompression and the re-establishment of coronal and sagittal balance.^2^ ^3^ Selecting the surgical plan for ASD is a challenge for spinal surgeons.^4–6^

Posterior long fixation and fusion from the thoracic spine to the sacrum is one of the most common surgical treatments for ASD.^7–9^ However, there is some debate regarding the most appropriate upper instrumented vertebra for thoracolumbosacral fusion.^10^ Suk has suggested that fusing the upper thoracic vertebrae (UTV) rather than T10 might decrease adjacent segment disease, whereas Madjetko has reported that patients might benefit from upper thoracic spinal fusion.

To the best of our knowledge, there is no standard guideline for whether the UTV or lower thoracic vertebrae (LTV) are better for ASD treatment. In this meta-analysis, we compared the peri-operative parameters, clinical and radiological outcomes, complications and need for revision between the UTV and LTV as the site of the upper instrumented vertebra for ASD.

## Materials and methods

This study was performed according to the preferred reporting items of the systematic review and meta-analyses (PRISMA) guidelines (see online supplementary checklist S1).^11^

10.1136/bmjopen-2016-012103.supp1 Supplementary checklist

### Search strategy

A comprehensive MEDLINE, Embase and Cochrane Library search was performed on 31 July 2016, by two independent authors (XF and XLS) using various combinations of the following search terms: ‘“proximal fusion level” or “upper instrumented vertebra” or “proximal junctional kyphosis”, or “upper instrumented thoracic vertebra” and “degenerative lumbar deformity”, or “adult lumbar deformity”, or “adult spinal deformity”, “degenerative lumbar scoliosis”, or “adult scoliosis”’. The search strategy developed for use with the MEDLINE database is shown in online supplementary table S1. Peer-reviewed articles reporting outcome measures for thoracolumbar and thoracolumbosacral instrumentation correction of ASD were collected. The reference lists of key articles were examined for eligible studies, and searches were performed with Google Scholar to avoid initial omissions.

10.1136/bmjopen-2016-012103.supp2 Supplementary table S1

### Inclusion criteria

All studies comparing the UTV and LTV as the upper instrumented vertebra for ASD were included. The inclusion criteria for the studies were as follows: (1) a minimum age of 18 years for all patients; (2) ASD, adult lumbar deformity or degenerative scoliosis as the primary indication for surgery; (3) a comparison of the UTV and LTV as the site of the upper instrumented vertebra for the treatment of ASD; and (4) a final postoperative follow-up of at least 12 months.

The following exclusion criteria were used: (1) case reports or case studies without comparisons; (2) data related to peri-operative parameters, clinical and radiological outcomes, complications and revisions that could not be extracted or calculated; and (3) a follow-up of <12 months. If multiple studies reported the same cohort of patients, only the most recent publication with the largest sample size was included.

### Data items and extraction

The data parameters were predetermined and reported in the reference literature. The data extraction was performed in two phases by two reviewers (XF and XLS) and subsequently assessed for consistency by a third reviewer (AMW). A standardised form was used that included the following items: (1) basic characteristics, such as patient sample size, year of publication, country of the study, age and gender descriptors, and final postoperative follow-up period; (2) peri-operative data, such as operative time and estimated blood loss; (3) clinical outcomes, such as the Scoliosis Research Society (SRS) pain level, self-image, function, mental health, subtotal, satisfaction, and total scores and the Oswestry disability index (ODI) score; (4) radiographic outcomes, including thoracic kyphosis (TK), thoracolumbar kyphosis (TLK), lumbar lordosis (LL), proximal junctional kyphotic angle, C7 sagittal vertical axis (C7SVA) and pelvic incidence; and (5) postoperative complications and revisions related to proximal junctional kyphosis (PJK), pseudarthrosis and hardware implant failure.

### Quality assessment of the included studies

The quality of the included studies was assessed based on the methodological index for non-randomised studies (MINORS).^12^ Twelve items were scored as ‘0’ (not reported), ‘1’ (reported but inadequate) or ‘2’ (reported and adequate). Two independent reviewers (XF and XLS) assessed the quality of the included studies.

### Statistical analysis

The data suitable for the meta-analysis were evaluated with STATA software (V.12.0; StataCorp, College Station, Texas, USA). The weighted mean difference (WMD) was calculated for continuous outcomes, and the relative risk (RR) was calculated for dichotomous outcomes. A random-effect model was used to perform the pooled analysis.^13–15^ Heterogeneity was defined if the χ^2^ test was <0.10 or the I^2^ test was >30%. If heterogeneity was observed, a further sensitivity analysis was involved to omit one study and evaluate whether the other results were significantly affected. The publication bias was analysed using Begg's and Egger's tests.

## Results

### Literature search

**Figure 1:**
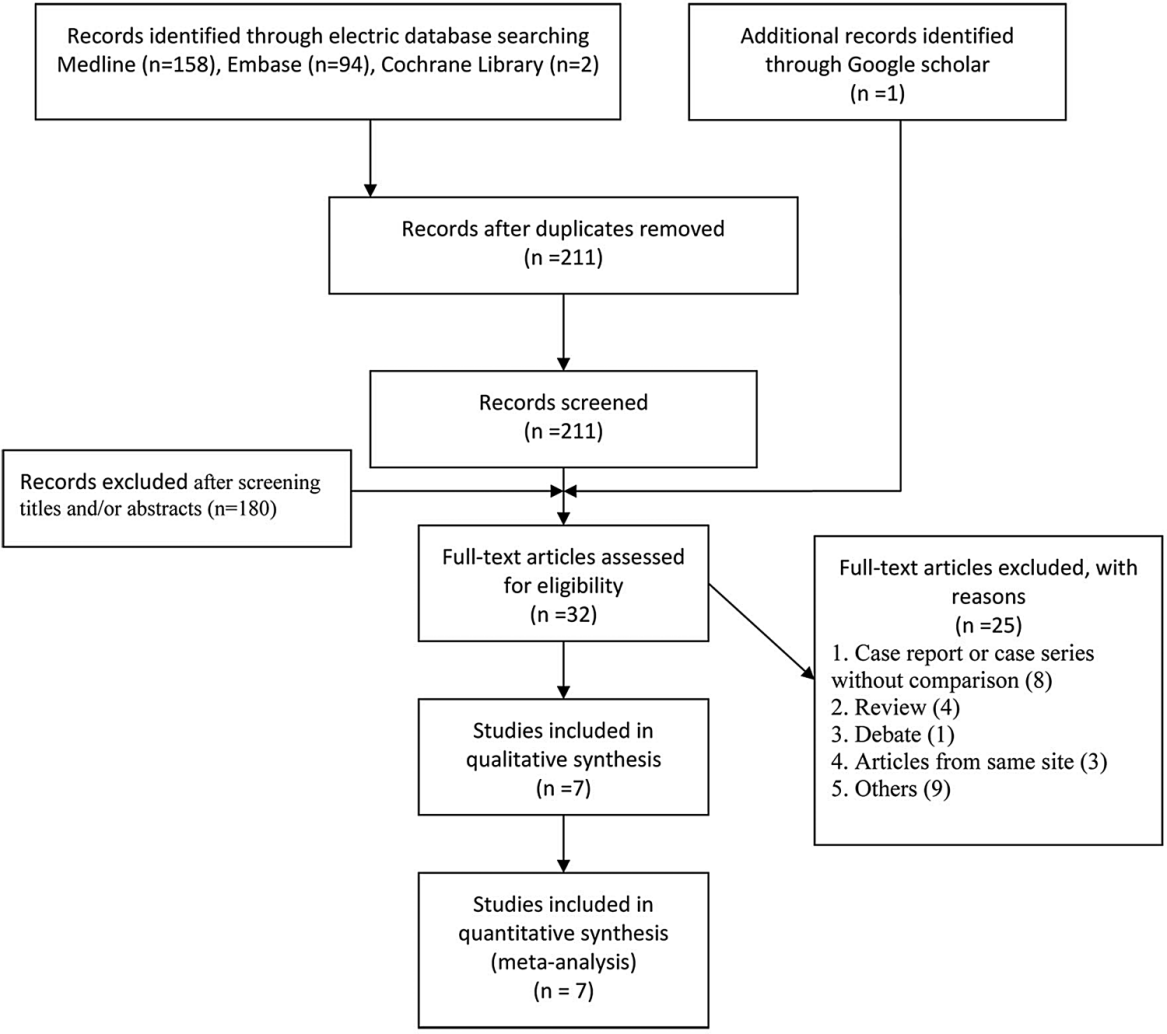
PRISMA flow diagram illustrating the selection of studies for inclusion. PRISMA, preferred reporting items of the systematic review and meta-analyses.

A total of 254 potential records were identified through MEDLINE (n=158), Embase (n=94) and the Cochrane library (n=2). After 43 duplicate articles were excluded, 211 articles were screened for titles and abstracts, which eliminated 180 articles. One article^16^ was added through a Google Scholar search. In total, 32 full-text articles were assessed for eligibility, and 25 were excluded because they were a ‘case report or case study without a comparison, a review article, a debate, an article from the same site as another included study, or other reasons’. Finally, seven non-random comparative studies^16–22^ were included in this meta-analysis. The included studies are shown in figure 1 (PRISMA flow diagram).

### Study characteristics

The characteristics of the seven non-randomised controlled trial (RCT) studies are listed in table 1.We did not find an RCT study comparing the UTV and LTV as the site of the upper instrumented vertebra for the treatment of ASD. Cho *et al*^17^ and Kim *et al*^20^ separated the proximal instrumented vertebrae data into three groups (T9–T10, T11–T12 and L1–L2). For this study, the T11–T12 and L1–L2 groups were included in the LTV data set. There were 232 patients in the UTV group and 322 in the LTV group; more than 2 years of follow-up data were available for both groups.

**Table 1:** Characteristics of the six included studies

Authors	Cho *et al*^17^	Scheer *et al*^18^	O'Shaughnessy *et al*^19^	Kim *et al*^20^	Fujimori *et al*^21^	Yagi *et al*^16^	Du *et al*^22^
Year	2013	2015	2012	2007	2014	2013	2016
Study design	Non-RCT	Non-RCT	Non-RCT	Non-RCT	Non-RCT	Non-RCT	Non-RCT
Intervention	UTV: T9–T10	UTV: T1–T6	UTV: T3–S	UTV: T9–T10	UTV: T1–T5	UTV: >T6	UTV: T9–11
	LTV: T11–L2	LTV: T9–L1	LTV: T10–S	LTV: T11–L2	LTV: T7–T12	LTV: <T8	LTV: L2–3
Number of participants	UTV: 22	UTV: 81	UTV: 20	UTV: 37	UTV: 31	UTV: 17	UTV: 24
	LTV: 29	LTV: 84	LTV: 38	LTV: 88	LTV: 49	LTV: 15	LTV: 19
Age (years)	UTV: 64.6±7.8	UTV: 60.3±11.3	UTV: 55.4±9.5	UTV: 51.9±11.3	UTV: 60±12	UTV: 48.7 (33–73)	–
	LTV: 64.6±5.2	LTV: 59.6±11.0	LTV: 55.9±8.5	LTV: 59.3±12.3	LTV: 62±10	LTV: 53.7 (33–76)	–
Gender	–	UTV: 15 M, 66 F	–	UTV: 1 M, 36 F	UTV: 4 M, 27 F	UTV: 3 M, 14 F	–
	–	LTV: 26 M, 58 F	–	LTV: 16 M, 72 F	LTV: 7 M, 42 F	LTV: 6 M, 9 F	–
Follow-up term	3.4±1.9	UTV: 2 years	UTV: 2.8±1.0	UTV: 4.7±4.4	UTV: 3.6±1.6	UTV: 9.4 (5–16)	2.3 (1.8–3)
		LTV: 2 years	LTV: 3.1±1.2	LTV: 4.5±2.6	LTV: 3.7±1.6	LTV: 7.9 (5–12)	

In the studies of Cho *et al* and Kim *et al*, the data for the LTV group represent the combination of the T11–T12 and L1–L2 groups in the original studies.

F, female; LTV, lower thoracic vertebra group; M, male; RCT, randomised controlled trial; UTV, upper thoracic vertebra group.

### Quality assessment

The methodological quality assessment of the seven included studies is summarised in table 2. Each of the seven studies clearly stated the aim of the study, and the participants were consecutive patients. The data in the study of O'Shaughnessy *et al*^19^ were collected prospectively, while in the other studies, the data were retrospectively collected. In the study of Kim *et al*,^20^ some patients did not finish the SRS score assessment; therefore, we assigned that study a score of ‘1’ for the ‘loss to follow-up less than 5%’ item. The scores ranged from 16 to 20, with a median value of 17.9. Publication bias was analysed using Begg's and Egger's tests; all of the p values were >0.05, and no publication bias was observed (see online supplementary table S2).

10.1136/bmjopen-2016-012103.supp3 Supplementary table S2

**Table 2:** Quality assessment of six included studies

Methodological item for non-randomized studies	Cho *et al*^17^	Scheer *et al*^18^	O'Shaughnessy *et al*^19^	Kim *et al*^20^	Fujimori *et al*^21^	Yagi *et al*^16^	Du *et al*^22^
1. A clearly stated aim	2	2	2	2	2	2	2
2. Inclusion of consecutive patients	2	2	2	2	2	2	2
3. Prospective collection of data	0	0	2	0	0	0	0
4. End points appropriate to the aim of the study	2	2	2	2	2	2	2
5. Unbiased assessment of the study end point	0	0	1	0	0	0	0
6. Follow-up period appropriate to the aim of the study	2	2	2	2	2	2	2
7. Loss to follow-up <5%	2	2	2	1	2	2	2
8. Prospective calculation of the study size	0	0	0	0	0	0	0
9. An adequate control group	2	2	2	2	2	2	2
10. Contemporary groups	2	2	2	2	2	2	2
11. Baseline equivalence of groups	2	2	1	1	1	2	2
12. Adequate statistical analyses	2	2	2	2	2	2	2
Total scores	18	18	20	16	17	18	18

### Operative time and estimated blood loss

**Figure 2:**
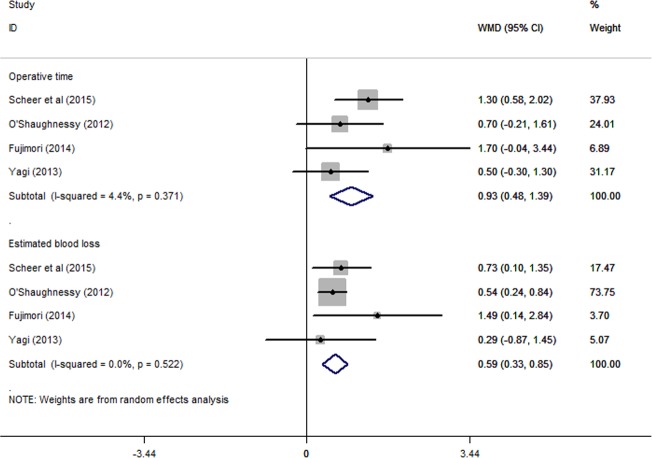
The meta-analysis results for operative time and estimated blood loss. The UTV group had a longer operative time (WMD: 0.93, 95% CI 0.48 to 1.39, p<0.05) and greater estimated blood loss (WMD: 0.59, 95% CI 0.33 to 0.85, p<0.05) than the LTV group, and both parameters showed statistically significant differences. LTV, lower thoracic vertebra; UTV, upper thoracic vertebra; WMD, weighted mean difference.

Four studies^16^ ^18^ ^19^ ^21^ reported the mean values and SDs for operative time and estimated blood loss. The meta-analysis showed that the UTV group had a longer operative time (WMD: 0.93, 95% CI 0.48 to 1.39, p<0.05) and a greater estimated blood loss (WMD: 0.59, 95% CI 0.33 to 0.85, p<0.05) compared with the LTV group, with both parameters showing a statistically significant difference (figure 2). No obvious heterogeneity was observed, with I^2^=4.4%, p=0.371 in the UTV group and I^2^=0.0%, p=0.522 in the LTV group.

### Clinical outcomes

**Figure 3:**
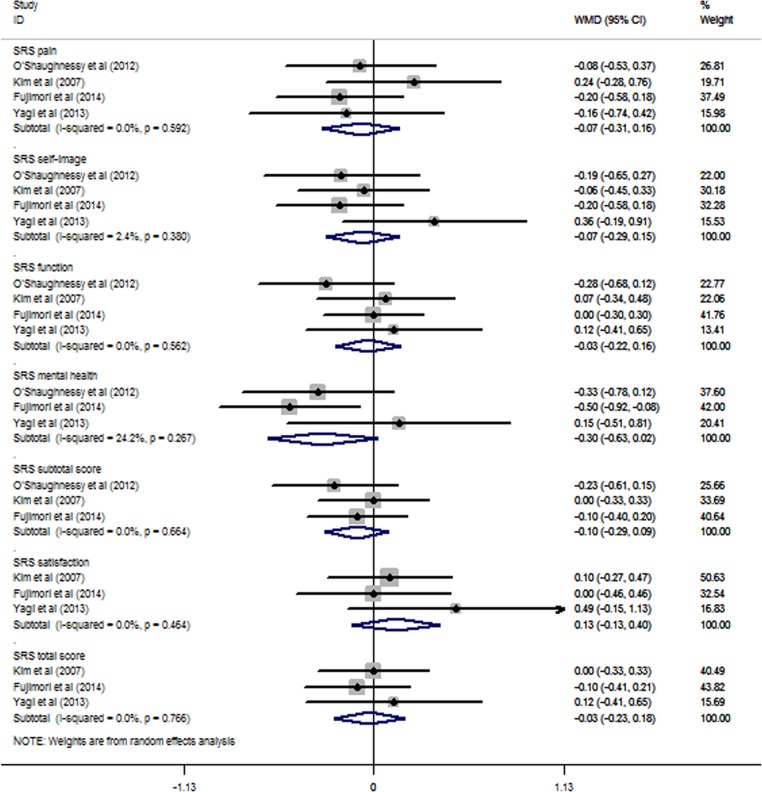
The meta-analysis results for the SRS outcomes. No statistically significant differences were found between the UTV and LTV groups. LTV, lower thoracic vertebra; SRS, Scoliosis Research Society; UTV, upper thoracic vertebra.

The studies of O'Shaughnessy *et al*,^19^ Kim *et al*,^20^ Fujimori *et al*^21^ and Yagi *et al*^16^ reported SRS scores, including pain (−0.07, 95% CI −0.31 to 0.16, p>0.05), self-image (−0.07, 95% CI −0.29 to 0.15, p>0.05), function (−0.03, 95% CI −0.22 to 0.16, p>0.05), mental health (−0.30, 95% CI −0.63 to 0.02, p>0.05), subtotal (−0.10, 95% CI −0.29 to 0.09, p>0.05), satisfaction (0.13, 95% CI −0.13 to 0.40, p>0.05) and total scores (−0.03, 95% CI −0.23 to 0.18, p>0.05). No statistically significant differences were found between the UTV and LTV groups (figure 3). The I^2^ of the SRS self-image score was 2.4%, and the I^2^ of the SRS mental health score was 24.2%; all others were 0.0%.

**Figure 4:**
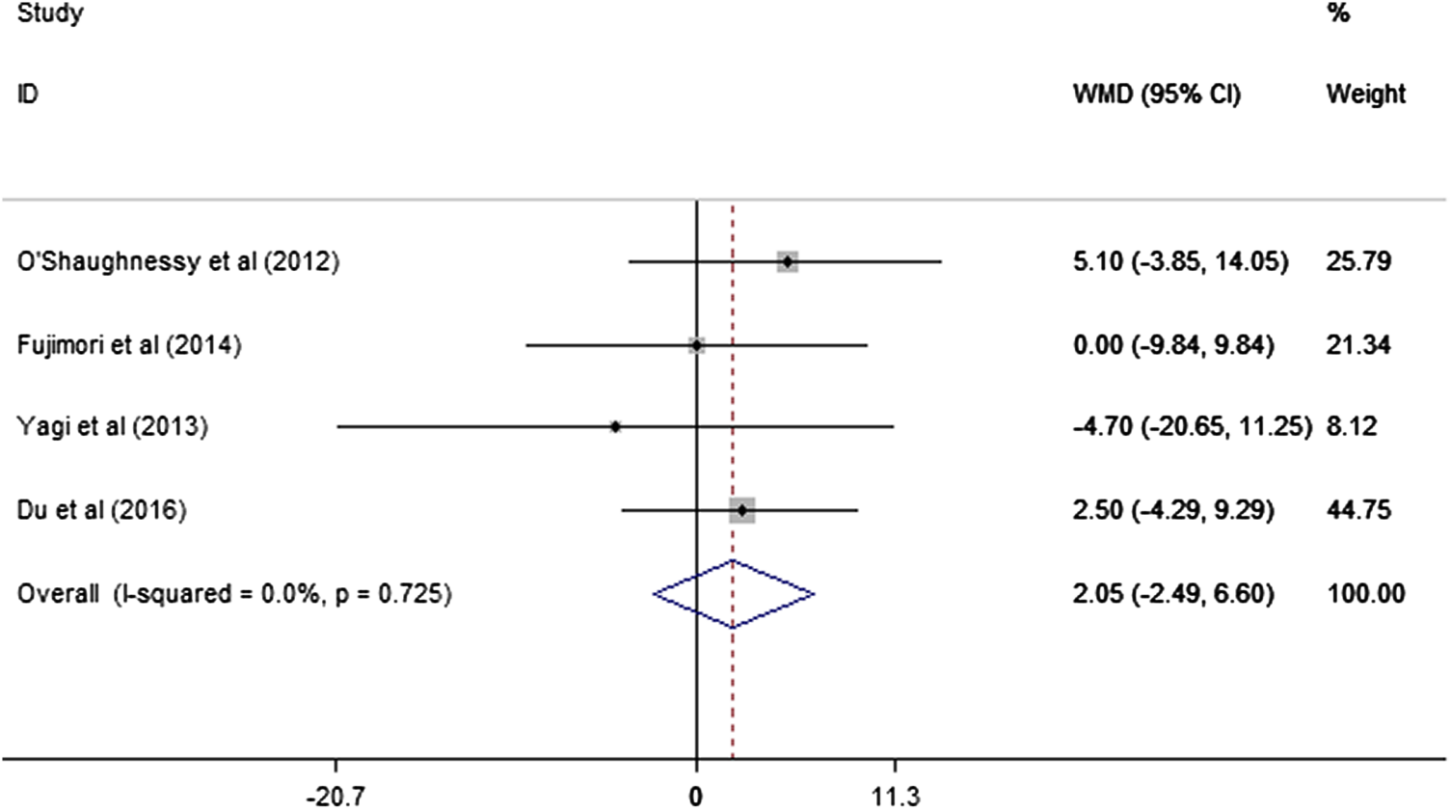
The meta-analysis results for the ODI score. No statistically significant difference between the UTV and LTV groups was found. LTV, lower thoracic vertebra; ODI, Oswestry disability index; UTV, upper thoracic vertebra.

The studies of O'Shaughnessy *et al*,^19^ Fujimori *et al*,^21^ Yagi *et al*^16^ and Du *et al*^22^ reported the ODI score results. The meta-analysis did not find a statistically significant difference between the UTV and LTV groups (WMD: 2.05, 95% CI −2.49 to 6.60), and no heterogeneity was observed (I^2^=0.0%, p=0.725; figure 4).

### Radiographic outcomes

**Figure 5:**
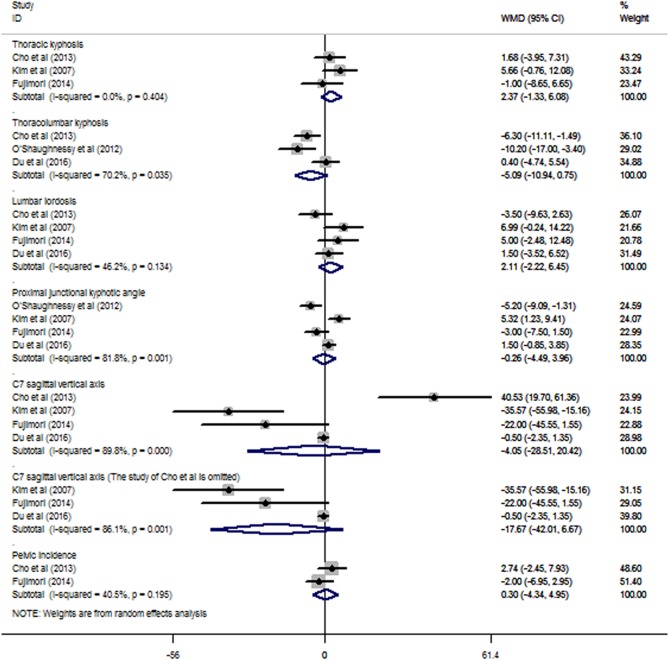
The meta-analysis of the radiographic outcomes showed no significant differences between the UTV and LTV groups in terms of TK, TLK, LL, PJK angle, C7SVA, and pelvic incidence. C7SVA, C7 sagittal vertical axis; LTV, lower thoracic vertebra; LL, lumbar lordosis; PJK, proximal junctional kyphotic; TK, thoracic kyphosis; TLK, thoracolumbar kyphosis; UTV, upper thoracic vertebra.

The meta-analysis of TK showed no significant difference between the UTV and LTV groups (WMD: 2.37, 95% CI 1.33 to 6.08), and no heterogeneity was observed (I^2^=0.0%, p=0.404; figure 5).

No significant differences were found in the meta-analyses of TLK, LL, PJK angle, C7SVA or pelvic incidence; all were observed to have heterogeneity, with I^2^=70.2%, 46.2%, 81.8%, 89.8% and 40.5%, respectively. The sensitivity analysis of the parameters revealed no significant changes in LL, PJK angle or pelvic incidence. The omission of Cho *et al*^17^ was found to significantly affect the C7SVA results (see online supplementary figure S1) and changed the WMD from −4.05 (95% CI −28.51 to 20.42) to −17.67 (95% CI −42.01 to 6.67; figure 5).

10.1136/bmjopen-2016-012103.supp4 Supplementary figure S1

### Complications and revision

**Figure 6:**
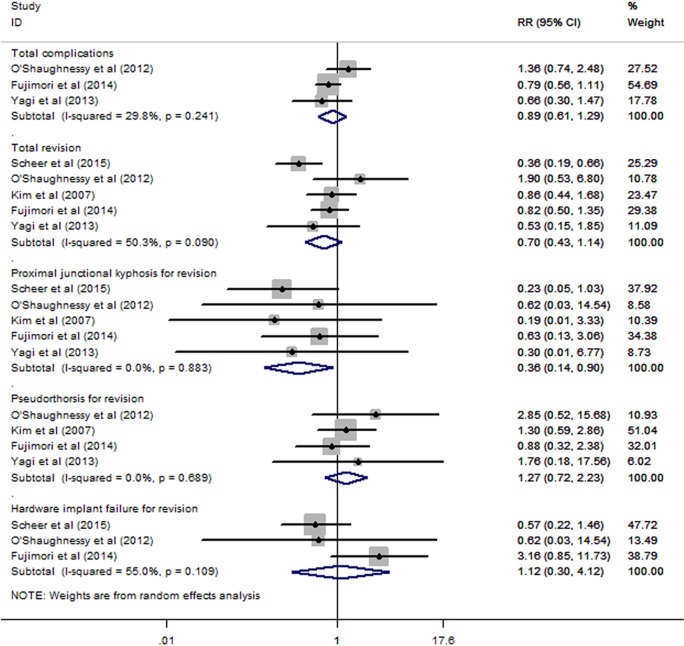
The meta-analyses of the total complications and revisions. No significant difference was found between the UTV and LTV groups for total complications and total revisions. The subgroup meta-analysis for revision surgery found that the UTV group had a lower risk of revision for PJK than the LTV group did (RR: 0.36; 95% CI 0.14 to 0.90); however, no significant differences in pseudarthrosis or hardware implant failure for revision were found (RRs: 1.27 (95% CI 0.72 to 2.23) and 1.12 (95% CI 0.30 to 4.12), respectively). LTV, lower thoracic vertebra; PJK, proximal junctional kyphotic angle; RR, relative risk; UTV, upper thoracic vertebra.

The meta-analyses of the total complications and total revisions revealed no significant difference between the UTV and LTV groups, with RRs of 0.89 (95% CI 0.61 to 1.29) and 0.70 (95% CI 0.43 to 1.14), respectively. The subgroup meta-analysis for revision surgery revealed that the UTV group had a lower risk of revision for PJK compared with the LTV group, with an RR of 0.36 (95% CI 0.14 to 0.90); no significant differences in pseudarthrosis or hardware implant failure for revision were found (RRs: 1.27 (95% CI 0.72 to 2.23) and 1.12 (95% CI 0.30 to 4.12), respectively; figure 6). Heterogeneity was observed in the meta-analyses of total revision and hardware implant failure for revision, with I^2^=50.3% and p=0.090 and I^2^=55.0% and p=0.109, respectively. The sensitivity analyses of these parameters showed no significant change when any one study was omitted (see online supplementary figure S1).

## Discussion

Degenerative spinal deformity is typically observed in patients over 60 years of age.^1^ ^23–25^ The symptoms of ASD vary from mild back pain without radiculopathy to severe back pain with radiculopathy, neurogenic claudication and even ambulatory intolerance.^26^ A positive radiographic test reveals coronal or sagittal imbalance or both, with or without spinal stenosis.^27^ Decreased LL and sagittal balance are the main causes of pain and functional loss.^28^ ^29^ The aims of surgical treatment are decompression, pain relief and the re-establishment of coronal and sagittal balance.^3^ ^30–32^

Posterior long fusion has been the primary surgical treatment for ASD;^33^ however, debate continues on the selection of the best proximal fused vertebra.^10^ ^34^ Using the LTV as the site of the upper instrumented vertebra has been reported to cause a high prevalence of PJK,^35–37^ and some surgeons suggest using the UTV for a longer fusion with the aim of reducing the incidence of PJK and the need for revision.

Scheer *et al*^18^ reported that ASD patients undergoing posterior fixation extending into the upper thoracic region have better sagittal spino-pelvic alignment and a lower risk of revision surgery; however, O'Shaughnessy *et al*^19^ and Kim *et al* 20 have reported conflicting results. In this meta-analysis, we synthesised data on complications and revision surgery, and no significant differences were found between the UTV and LTV groups. A further subgroup analysis of the various reasons for revision surgery was performed and indicated that the UTV group had a significantly lower risk of revision because of PJK (figure 6). The T11–L2 segment has always been regarded as the junctional spinal segment, and the T1–T10 segment is supported by the true ribs, whereas the T11–T12 segment has floating ribs without costosternal articulation. The biomechanics differ significantly between the rigid thoracic spine and the flexible lumbar spine in the T11–L2 region. This region has been reported as having a high incidence of fractures and kyphosis.^38^ ^39^ In addition, in the studies of Cho *et al*^17^ and O'Shaughnessy *et al*,^19^ the TLK was higher in the LTV group than in the UTV group; this finding supports the possibility that patients with postoperatively higher TLK are more likely to develop PJK and suggests that posterior fixation extending into the upper thoracic region could maintain sagittal alignment in the thoracolumbar region. Hyun *et al*^40^ reported that PJK patients had lower thoracolumbar muscularity and that lower thoracolumbar muscularity may induce higher TLK, resulting in a higher risk of PJK.

Although the UTV group had a decreased incidence of revision surgery for PJK, several deficiencies necessitated revisions. O'Shaughnessy *et al*^19^ reported that eight patients underwent revision surgery for the following reasons: PJK (one patient), pseudarthrosis (five patients) and pedicle fracture and iliac screw removal (two patients). Kim *et al*^20^ reported that 31 patients underwent revision surgery for PJK (5 patients) or pseudarthrosis (21 patients). Fujimori *et al*^21^ reported that 7 of 38 revision surgeries were for PJK and 14 of 31 were for pseudarthrosis. Pseudarthrosis is the cause of the highest proportion of revision surgeries, and the subgroup meta-analyses for revision surgery due to pseudarthrosis and hardware implant failure showed no difference in the RR between the UTV and LTV groups. This might explain why no significant difference was found in total revision surgery between the two groups.

Posterior fixation extending into the upper thoracic region results in a longer operative time and greater intraoperative blood loss. In this meta-analysis, the operative time of the UTV group was significantly longer than that of the LTV group (WMD: 0.93, 95% CI 0.48 to 1.39), and the UTV group had a greater estimated blood loss than did the LTV group. Most ASD patients are elderly,^24^ ^25^ and the increased number of fused segments might increase the implant cost and lengthen postoperative recovery. Individual surgical endurance levels and life expectancy^41^ should be considered before making surgical decisions in these cases.

Another limitation is that there was no consistent definition of which vertebra constitutes UTV and which constitutes LTV. Clinically, the biomechanical transition region of the T11–L2 segment has always been regarded as the separating line by most surgeons; sites above this region were regarded as UTV, and those below it were considered LTV. To clarify to the readers how the UTV and LTV were determined in the studies included in this meta-analysis, the UTV and LTV designations for all of the included studies are listed in table 1. The differences in these designations may have introduced heterogeneity into the present meta-analysis.

### Implications for future research and conclusions

Current evidence shows that long posterior fixation extending into the upper thoracic region provides better correction of TLK and reduces the incidence of revision surgery related to PJK. Increasing the operative level results in longer operative times and a higher estimated blood loss. The UTV and LTV groups had similar outcomes in terms of SRS scores, ODI scores, total complications and the total number of revision surgeries. This initial analysis indicates that extending fixation to the upper thoracic region is appropriate in patients who are likely to develop PJK following the initial scoliosis correction. Additional high-quality studies (RCTs with larger sample sizes) using the same surgical intervention protocol and follow-up time are needed to decrease heterogeneity and to confirm the reported effects.
