# Supplementary material for: Long fusion correction of degenerative adult spinal deformity and the selection of the upper or lower thoracic region as the site of proximal instrumentation: a systematic review and meta-analysis
Source: BMJ Open. 2016 Nov 15;6(11):e012103. doi: 10.1136/bmjopen-2016-012103 (PMC5128941; doi:10.1136/bmjopen-2016-012103)
Supplement: Supplementary table S1 [file bmjopen-2016-012103supp_Table1.pdf]

Table S1: The developed search strategy performed in database of Medline.

No	Search terms
#1	proximal fusion level
#2	upper instrumented vertebra
#3	proximal junctional kyphosis
#4	upper instrumented thoracic vertebra
#5	#1 or #2 or #3 or #4
#6	degenerative lumbar deformity
#7	adult lumbar deformity
#8	adult spinal deformity
#9	degenerative lumbar scoliosis
#10	adult scoliosis
#11	#6 or #7 or #8 or #9 or #10
#12	#5 and #11
