# Supplementary material for: Long fusion correction of degenerative adult spinal deformity and the selection of the upper or lower thoracic region as the site of proximal instrumentation: a systematic review and meta-analysis
Source: BMJ Open. 2016 Nov 15;6(11):e012103. doi: 10.1136/bmjopen-2016-012103 (PMC5128941; doi:10.1136/bmjopen-2016-012103)
Supplement: Supplementary table S2 [file bmjopen-2016-012103supp_Table2.pdf]

Table S2: The results of publication bias analyzed by both Begg's and Egger's test.

Parameters	*P* value by Begg's test	*P* value by Egger's test
Operative time	0.308	0.474
Estimated blood loss	1.000	0.700
SRS pain	0.734	0.502
SRS self-image	0.308	0.219
SRS function	0.308	0.888
SRS mental health	0.296	0.106
SRS subtotal score	1.000	0.595
SRS satisfaction	1.000	0.447
SRS total score	0.296	0.353
ODI	0.308	0.296
Total complications	1.000	0.841
Total revision	0.806	0.705
Proximal junctional kyphosis for revision	0.462	0.919
Pseudorthorsis for revision	0.734	0.412
Hardware implant failure for revision	1.000	0.877

Note: all of the P value > 0.05, none of publication bias was observed.
