# Supplementary material for: Long fusion correction of degenerative adult spinal deformity and the selection of the upper or lower thoracic region as the site of proximal instrumentation: a systematic review and meta-analysis
Source: BMJ Open. 2016 Nov 15;6(11):e012103. doi: 10.1136/bmjopen-2016-012103 (PMC5128941; doi:10.1136/bmjopen-2016-012103)
Supplement: Supplementary figure S1 [file bmjopen-2016-012103supp_Figure.pdf]

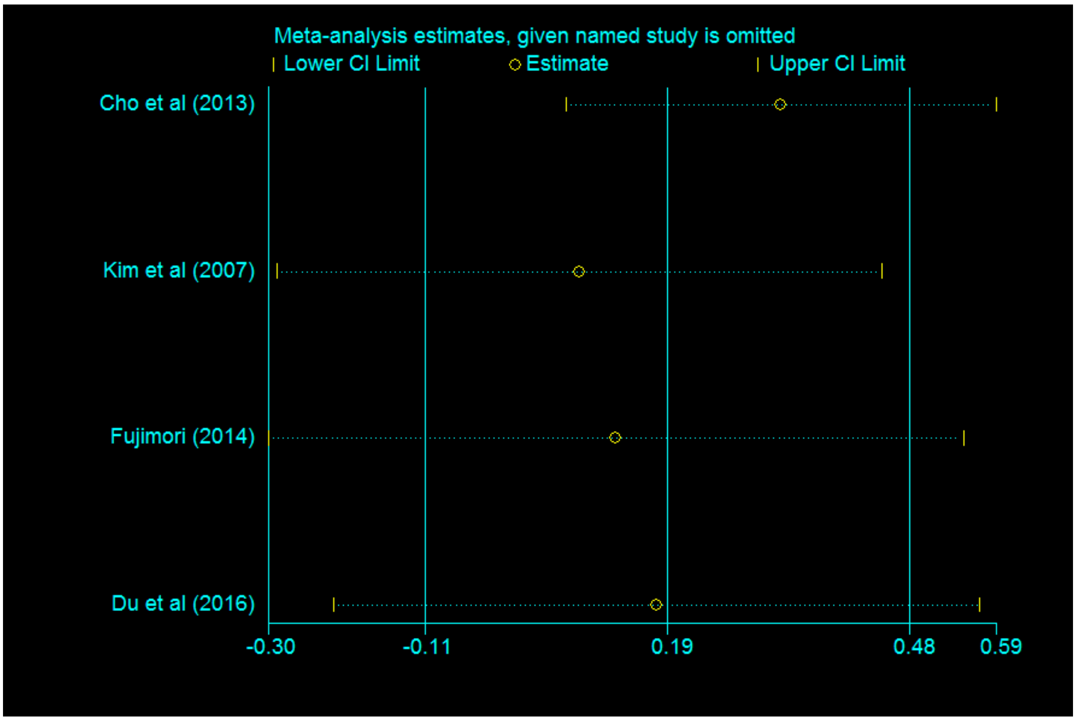

Figure S1: A: The sensitivity analysis of the lumbar lordosis.

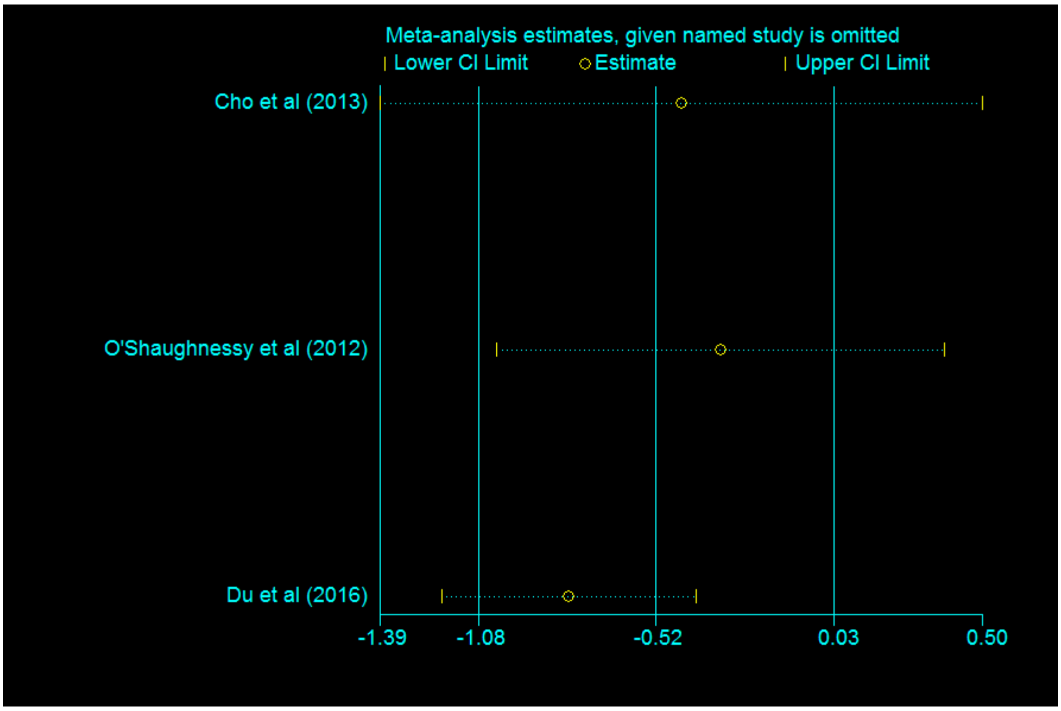

Figure S1: B: The sensitivity analysis of thoracolumbar kyphosis.

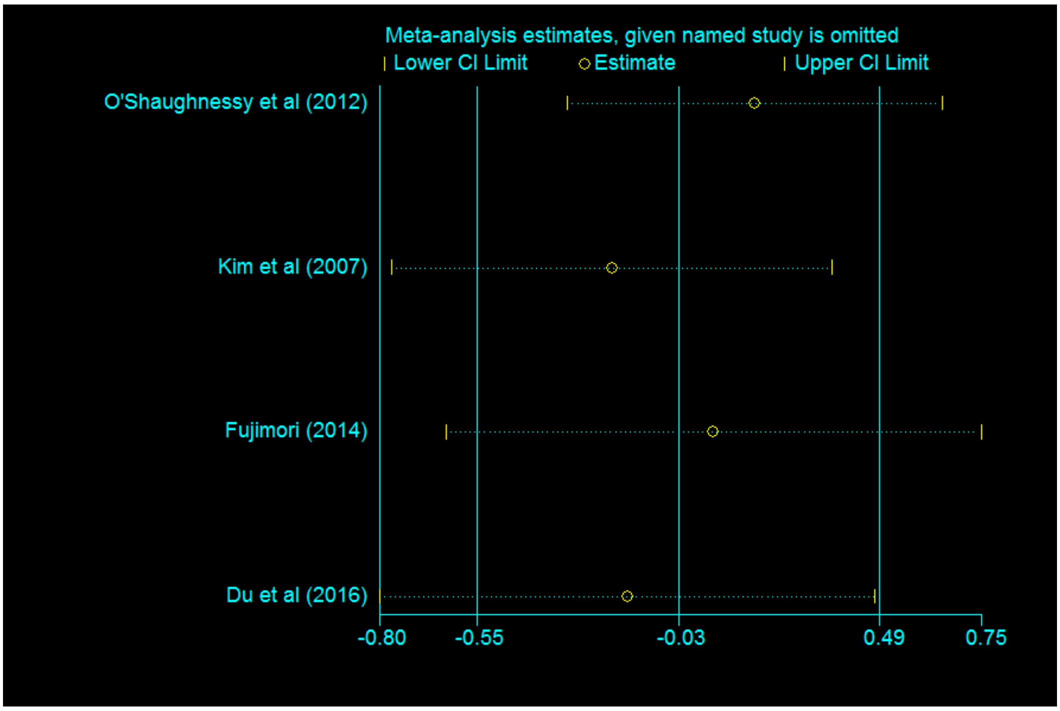

Figure S1: C The sensitivity analysis of the proximal junctional kyphotic angle.

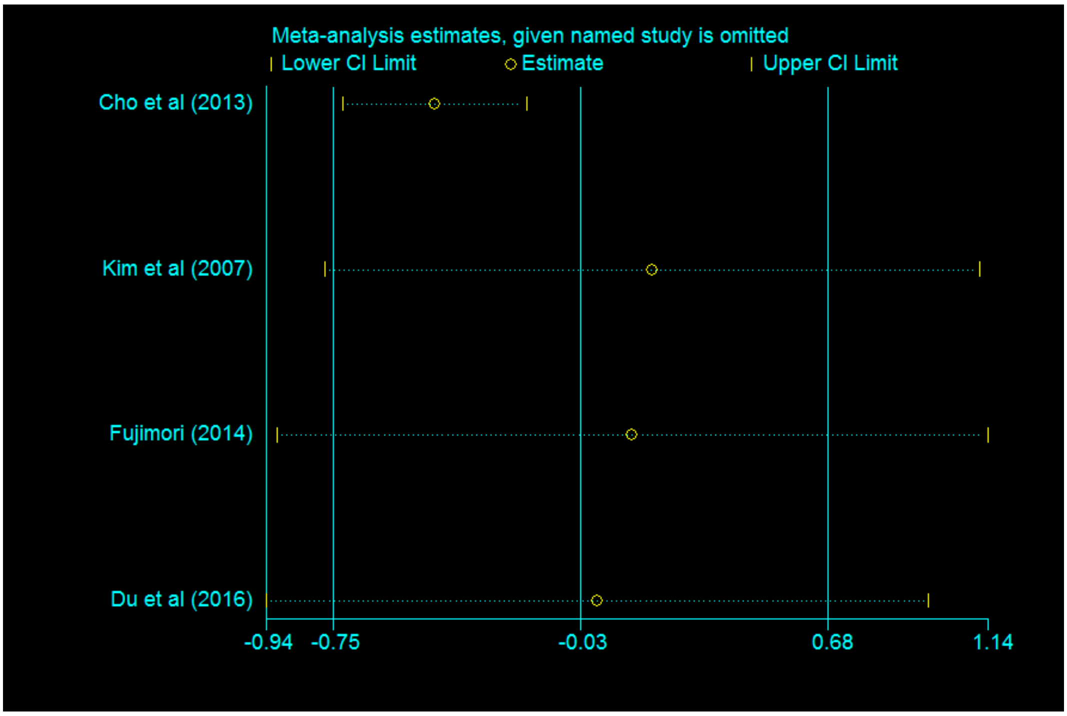

Figure S1: D: The sensitivity analysis of the C7 sagittal vertical axis. The estimated effect size was changed significantly after the study o f Cho et al was omitted.

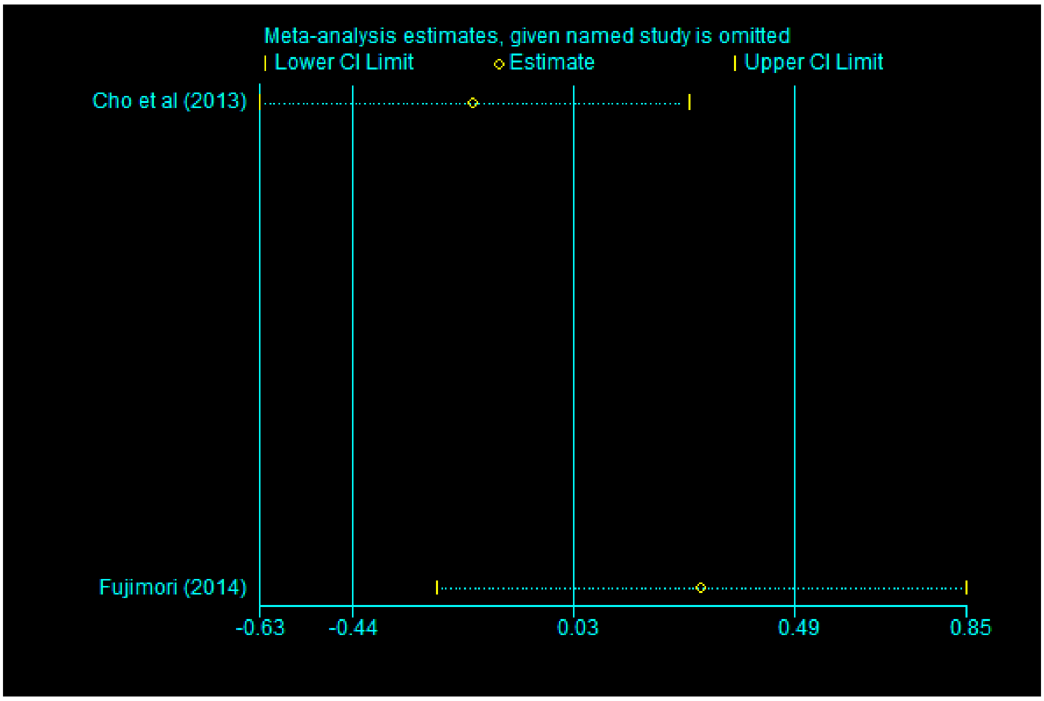

Figure S1: E: The sensitivity analysis of the pelvic incidence.

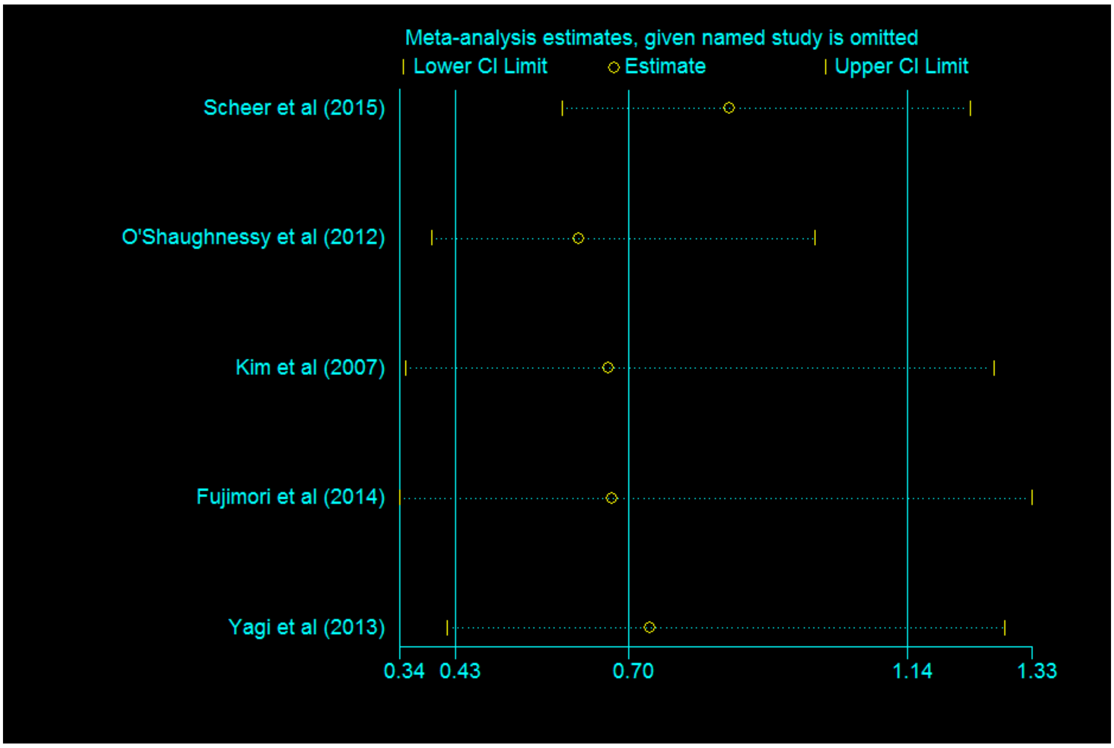

Figure S1: F: The sensitivity analysis of the total revision.

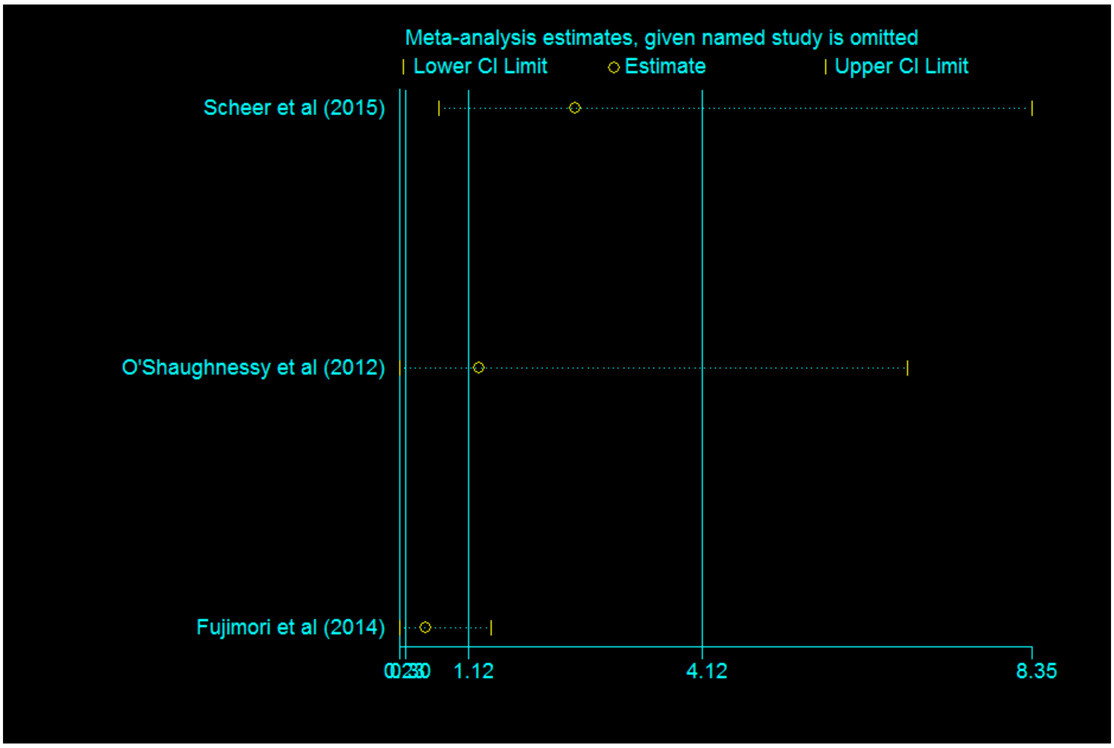

Figure S1: G: The sensitivity analysis of the hardware implant failure revision.
